# Health condition of Afghan refugees residing in Iran in comparison to Germany: a systematic review of empirical studies

**DOI:** 10.1186/s12939-023-01832-7

**Published:** 2023-01-21

**Authors:** Parisa Rahimitabar, Alexander Kraemer, Kayvan Bozorgmehr, Fatemeh Ebrahimi, Amirhossein Takian

**Affiliations:** 1 FlüGe Research Project (Refugee Health), School of Public Health, Bielefeld University, P.O. Box 100131, 33501 Bielefeld, Germany; 2 FlüGe Research Project, School of Public Health, Bielefeld University, Bielefeld, Germany; 3 Department of Population Medicine and Health Services Research, School of Public Health, Bielefeld University, P.O. Box 100131, 33501 Bielefeld, Germany; 4 Section for Health Equity Studies & Migration, University Hospital Heidelberg, Heidelberg, Germany; 5 School of Public Health, Bielefeld University, P.O. Box 100131, 33501 Bielefeld, Germany; 6 Department of Global Health and Public Policy, School of Public Health, Tehran University of Medical Sciences (TUMS), P.O. Box 1417613151, Tehran, Iran; 7 Department of Health Management, Policy & Economics, School of Public Health, TUMS, Tehran, Iran; 8 Heath Equity Research Center (HERC), TUMS, Tehran, Iran

**Keywords:** Health, Afghan refugees, Systematic review, Mixed-method, Germany, Iran

## Abstract

**Background:**

The re-emerging dominance of the Taliban in Afghanistan in 2021 caused a new wave of Afghan refugees heading Iran and neighboring countries. Iran in the Middle East and Germany in Europe are two major host countries to the largest populations of Afghan refugees. In both countries, several studies have been done to assess the health condition of refugees.

**Objectives:**

To systematically review the existing literature to identify similarities and differences of health conditions of Afghan refugees living in the two countries, and to synthesize evidence on the health status and health care access of these populations.

**Methods:**

Related electronic databases and grey literature of Iran and Germany on the health of Afghan refugees were scanned and searched up for the period 2000–2020. Key terms were formed by combining “Afghan refugees or immigrants or populations or asylum seekers”, “Physical or mental health”, “Healthcare service or access or use”, “Iran or Germany”. Empirical studies were considered if they contained samples of Afghan refugees with particular outcomes for Afghans. Results were categorized for both countries in the three main areas of physical health, mental health, and access/use of healthcare services.

**Results:**

Nine hundred twenty-two documents were extracted, of which 75 full-texts were finally reviewed. 60 documents belonged to the health condition of Afghan refugees residing in Iran including 43 in physical health, 6 in mental health, 8 in healthcare access and use, and 3 in multiple aspects of health, and 15 belonged to Germany including 7 in physical health, 4 in mental health, 2 in healthcare access and use, and 2 in multiple aspects of health. A less explicit evaluation of the overall health condition of Afghan refugees was observable, particularly for Germany. While matches on the study subject exist for both countries, in comparison to Germany, we extracted more quantitative and qualitative health studies on Afghan refugees of the mentioned areas from Iran. German health studies were rare, less qualitative, and more on the health condition of diverse refugee groups in general.

**Conclusions:**

Wide gaps and unanswered questions related to mental health and overall health status of the Afghan refugee population are observable, especially in Germany. Our systematic review identified the gap in evidence, which we would recommend to bridge using a wider lens to comprehensively assess the overall condition of refugees considering associations between health and socio-economic and cultural determinants instead of a one-dimensional approach. Further, within health studies on refugee populations, we recommend stratification of results by the country of origin to capture the within-group diversity among refugees with different countries of origin.

## Background

Afghanistan is located in the south center of Asia and borders Iran with a population of more than 39 million. The country has been suffering from various issues including weak governance, established terrorism, civil war, inequal resources distribution, a high proportion of the population living below the poverty line, lack of a comprehensive social protection system, and illegal production and trade of opium products [1], all of which have compromised the health condition of Afghans. For instance, Afghanistan is one of the most dangerous places for children and mothers, access to a hospital or health facilities is difficult, has one of the highest infant mortality rates in the world, and many Afghan females die from preventable pregnancy-related causes [2]. As a result, for the period of over four decades, it has been one of the major epicenters of producing refugees and displaced people worldwide. The re-emerging dominance of the Taliban in Afghanistan in 2021 caused a new wave of Afghans heading Iran and neighboring countries. Alone in 2021, 667,903 Afghans have been internally displaced in Afghanistan. Current situation caused a new wave of Afghan asylum seekers heading Iran and neighboring countries. While most Afghan refugees and asylum seekers have settled in Iran [3, 4], Germany has been one of the major European countries of their destination [5, 6].

Since the beginning of the year 2021, the United Nations High Commissioner for Refugees (UNHCR) identified 22,086 Afghans who newly arrived in Iran [4]. Short-term and periodic migration of Afghans to Iran was common before the 1979 Iranian Islamic revolution and it was mainly due to poverty, drought, and economic reasons. However, large-scale migration of Afghan refugees during the last three decades was the consequence of political crisis and war in Afghanistan [3]. Today (November 2021), almost 1 million registered Afghan refugees and approximately 2.6 million unregistered Afghans live in Iran who are given different levels of protection and access to basic services depending on their registration [4]. For decades, Iran’s Ministry of Health & Medical Education (MoHME) together with domestic and foreign donors, Non-Governmental Organizations (NGOs), the UNHCR, and Iran’s Bureau for Aliens and Foreign Immigrants Affairs (BAFIA) have been planning and combining financial resources to provide educational and healthcare services with the priority given to the registered Afghan refugees and more vulnerable Afghans [7]. However, healthcare needs are high, and resources are not enough to cover each of the most vulnerable registered refugees.

Germany is the fifth largest host country of refugees in the world hosting 1.2 million refugees [8]. It has experienced various forms of immigration over the decades. The continuous migration of asylum seekers and refugees increased to a peak in the early 1990s as well as in the years 2015 and 2016. The majority of recent refugees and asylum-seekers in Germany come from Syria, Afghanistan, and Iraq and they are relatively young and predominantly male [8, 9]. According to UNHCR, around 148,000 Afghan refugees are currently residing in Germany [8]. The refugee migration has brought several challenges to this country including responding to the needs for emergency housing, handling the language barrier, and facilitating refugees’ longer-term integration [10]. In addition, it has also affected the German health system, public health, and health promotion. The health system is under pressure to provide asylum-seekers and refugees with access to necessary medical care and services [11].

In Iran and Germany, as refugees’ major hosts in Asia and Europe, several studies have been done to assess the health condition of refugee populations residing in the two countries. The current review questioned the types and approaches of the so far performed refugees’ health studies on the Afghan population residing in both countries to assess the studies’ performances and success in estimating the overall health conditions of these vulnerable populations. Further, it aimed to review the existing literature for similarities and differences of Afghan refugees’ health conditions as one of the main refugee populations living in the two countries, and to assess and contrast the physical and mental health conditions and healthcare access and use.

## Methods

We followed the PRISMA guidelines to conduct this mixed-method systematic review [12]. To answer the questions a structured review of the relevant literature was undertaken by following the search strategy and selection criteria below.

### Search strategy

Literature on the health condition of Afghan refugees residing in Iran and Germany was searched and extracted from web-based databases including PubMed, EBSCO, Emerald, Elsevier, ProQuest, EMBASE, Science Direct, Scopus, Cochrane Library, and Google scholar from the year 2000 until the end of 2020. Most of the empirical studies that contained samples of Afghan refugees with particular outcomes for Afghans had been conducted over the last twenty years in the two countries. Further, the Iranian databases including SID, Magiran, IranMedex, and Irandoc were searched for relevant locally published articles. Key terms were formed by combining “Afghan refugees or immigrants or populations or asylum seekers”, “Physical or mental health”, “Healthcare service or access or use”, “Iran or Germany”. Based on our search strategy, only peer-reviewed articles with one of the searched keywords either in their title or in their abstract were extracted.

### Study inclusion and exclusion criteria

Articles were included if at least an area of ​​physical or mental health or healthcare access or utilization were addressed and if the articles were written in English, German, or Persian (Farsi) language. We defined Afghan refugees as all individuals from Afghanistan who migrated to Iran or Germany in search of legal protection regardless of the motives of seeking protection. All original and review articles were included in this systematic review including systematic, narrative, and literature reviews, mixed-method, quantitative (cohorts, case–control, cross-sectional, surveys, studies using secondary data, longitudinal, experimental), qualitative (in-depth or semi-structured interviews, focus groups, ethnographies, participatory action research), empirical and epidemiological studies, and reports. This approach of inclusion is suggested by a published protocol for conducting systematic reviews on the health status of and healthcare provision to asylum seekers in Germany [13]. Letters to the editor, editorials, policy documents, book chapters, and dissertations were excluded. Studies that reported on Afghan refugees residing in countries other than either Iran or Germany or studies addressing refugee health in general with unclear population ethnicity were not included. The size of the sample population, their legal status (registered/unregistered), gender, and age specifications were not considered as selection criteria.

### Study quality assessment

As the current review considered a wide range of study designs for inclusion, several quality appraisal tools were used to assess and evaluate the eligible articles for the final review. For this intention, the Public Health Practice Project (EPHPP) appraisal tool was used for the articles with quantitative methods, the Critical Appraisal Skills Program (CASP) checklist for the qualitative ones, A MeaSurement Tool to Assess systematic Reviews (AMSTAR) for normal systematic reviews and the Mixed Methods Appraisal Tool (MMAT) was applied to assess systematic reviews that were including mixed method studies. Articles that did not meet the quality appraisal tools requirements were excluded from the final review.

## Results

**Fig. 1:**
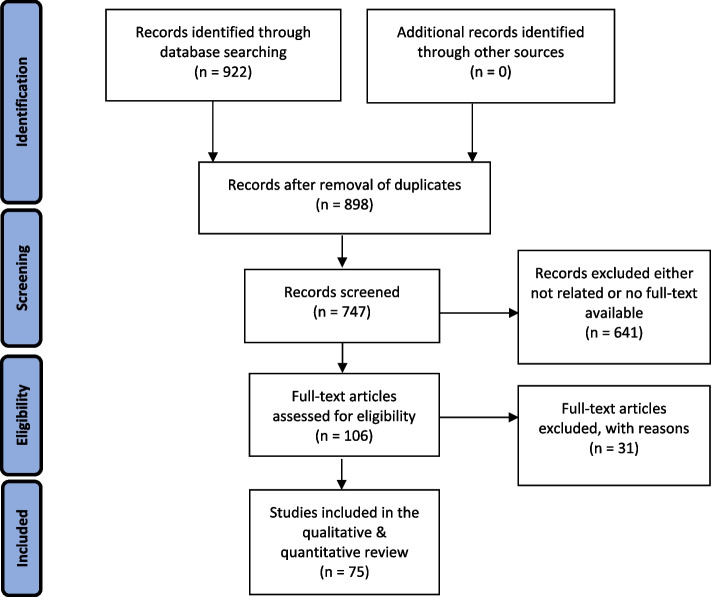
PRISMA flowchart of screened and included studies

Following our developed search strategy, 922 results were extracted, imported to the Zotero software, and categorized based on all inclusion and exclusion criteria. All potentially relevant titles were screened for inclusion. Two raters (PR^1^ and FE^4^) then independently reviewed the abstracts of retained articles to further ensure the proper inclusion. Discrepancies in decisions related to the inclusion or exclusion of studies were reconciled through mutual agreement. After initial assessments, 175 duplications, letters to the editor, editorials, interviews, policy documents, book chapters, and dissertations were excluded and then the abstracts and titles of the remaining 747 articles were reviewed. In the next stage, 618 studies were excluded as they were unrelated to the purpose of this study. Besides, 23 more studies were excluded due to the lack of full-text availability. Therefore, 106 studies were subjected to a full-text review. The full-texts of the 106 articles were evaluated and again 31 studies that either did not match the aim of this systematic review or did not fulfill the quality appraisal standards, were excluded. Finally, 75 eligible articles with various study approaches were reviewed among which 60 studies were related to Iran and 15 studies were related to Germany. Figure 1 shows the PRISMA diagram of this review which provides an outlay for the study selection process. Table 1 summarizes the characteristics of Afghan refugees residing in Iran and Germany. Table 2 reports the areas of the reviewed studies and numbers for both Iran and Germany, and Table 3 summarizes the characteristics of the reviewed studies.

**Table 1:** Characteristics/background comparison of Afghan refugees residing in Iran and Germany

Countries Dimensions	Iran	Germany
Population of Afghan refugees	3.6 million, first asylum was 1979	148,000; major first asylums were 2014
Age of Afghan refugees	older/second-generation refugees	younger
Gender of Afghan refugees	balanced distribution	predominantly male
Language of host country	same/similar (Farsi and Pashto)	very different (German)
Religion of host country	mostly Muslim	mainly Christian
Culture of host country	similar	very different
Modernity of host country	lower modernity, easier employment	higher modernity, harder to meet job requirements (e.g., education levels & specialties)
Economy of host country	weaker – as developing country struggling with several economic sanctions	stronger – industrial/developed country
Health system of host country	centralized, same refugee regulations/policies all over the country	decentralized, different refugee regulations/policies among provinces (e.g., health vouchers versus health electronic cards)

**Table 2:** Categorized findings based on areas and methods of the reviewed studies

Iran	
Physical health	43
Mental health	6
Healthcare access & use	8
Multiple aspects of health	3
*Total:*	60
Quantitative	40
Qualitative	8
Mixed-methods	2
Epidemiological	2
Systematic review	5
Narrative review	1
Literature review	1
Report	1
Germany	
Physical health	7
Mental health	4
Healthcare access & use	2
Multiple aspects of health	2
*Total:*	15
Quantitative	11
Qualitative	1
Mixed-methods	1
Epidemiological	1
Report	1

**Table 3:** Characteristics of the reviewed studies (categorized by the study area)

	First author	Publication year	Area	Location	Method	Sample size	Sample size of Afghans
*Iran: physical health*							
1	Sharifi [14]	2020	Pregnancy	Tehran	Quantitative, cross-sectional	280	the same
2	Mohammadi [15]	2017	Maternal-Near-Miss (MNM)	Tehran	Mixed-methods (audit study + interviews)	76	22
3	Mohammadi [16]	2016	MNM	Tehran	Quantitative, case–control	82 cases 1,024 controls	22 cases 150 controls
4	Rezaeian [17]	2014	Neonatal	Rafsanjan	Quantitative, cross-sectional	5,925	393
5	Abbasi Shahvazi [18]	2015	Fertility	Iran	Quantitative, census	230,054	14,741
6	Sadeghipour Roudsari [19]	2006	Reproductive health	Tehran	Quantitative, cross-sectional	478	231
7	Tober [20]	2006	Family planning	Isfahan	Qualitative, ethnographic	101	the same
8	Tober [21]	2007	Public health	Isfahan	Qualitative, interviews	101	64
9	Nateghi Rostami [22]	2013	Leishmaniosis	Qom	Quantitative, cross-sectional	849	100
10	Mohammadi Azni [23]	2010	Leishmaniosis	Damghan	Quantitative, cross-sectional	465	20
11	Otoukesh [24]	2012	Disease referrals	Iran	Quantitative, cross-sectional	23,152	the same
12	Pakravan Charvadeh [25]	2020	Food security	Tehran	Quantitative, cross-sectional	317	the same
13	Maarefvand [26]	2016	Food security	Tehran	Quantitative, cross-sectional	150	the same
14	Abdollahi [27]	2015	Food security	Pakdasht	Quantitative, cross-sectional	414	the same
15	Omidvar [28]	2013	Food security	Tehran & Mashhad	Quantitative, cross-sectional	310	the same
16	Rezaeian [29]	2009	Nutrition	Tehran	Quantitative, cross-sectional	606	the same
17	Fallah [30]	2016	Enterobacteriaceae	Iran	Quantitative, correlational	2,500	1,112
18	Pourhossein [31]	2015	Infectious diseases	Iran	Systematic review & meta-analysis	-	-
19	Barati [32]	2010	Cholera	Karaj	Quantitative, case–control	54	4
20	Rajabali [33]	2009	Communicable diseases	Iran & Pakistan	Narrative review	-	-
21	Izadi [34]	2004	Crimean-Congo fever	Sistan & Baluchestan	Quantitative, case–control	24 cases 300 controls	5
22	Behzadi [35]	2014	Hepatitis B	Fars	Quantitative, cross-sectional	all Afghans in the province	64
23	Fathimoghaddam [36]	2011	Hepatitis B	Mashhad	Quantitative, cross-sectional	652	61
24	Naseh [37]	2019	Substance use	Tehran, Mashhad, Isfahan, Shiraz & Kerman	Mixed-methods	Quantitative: 2,034 Qualitative: 8	the same
25	Noori [38]	2016	Drugs & HIV	Tehran	Quantitative, cross-sectional	81	the same
26	Khosravi [39]	2018	HIV	Karaj	Quantitative, randomized controlled trial	61	the same
27	Jahanbakhsh [40]	2013	Drugs & HIV	Tehran, Shiraz & Kermanshah	Quantitative, cross-sectional	10	the same
28	Jabbari [41]	2011	HIV	Tehran	Quantitative, survey	477	the same
29	Norouzinejad [42]	2016	Malaria	Iran	Epidemiological	-	-
30	Youssefi [43]	2011	Malaria	Sarbaz	Quantitative, survey	1,464	49
31	Jimma [44]	2017	TB	Iran & its neighbors	Systematic review & meta-analysis	-	-
32	Rahmanian [45]	2016	TB	Jahrom	Quantitative, descriptive-analytical	114	36
33	Nasiri [46]	2014	TB	Iran	Quantitative, cross-sectional	4,950	179
34	Ramazanzadeh [47]	2009	TB	Iran	Quantitative, cross-sectional	523	185
35	Farnia [48]	2008	TB	Tehran	Quantitative, survey	258	59
36	Velayati [49]	2006	TB	Tehran	Quantitative, survey	1,742	668
37	Ramazanzadeh [50]	2006	TB	Iran	Quantitative, cross-sectional	345	87
38	Kadivar [51]	2007	TB	Fars	Epidemiological	1,026	371
39	Farnia [52]	2006	TB	Tehran	Quantitative, survey	1,074	668
40	Moghani [53]	2010	Kidney transplantation	Iran	Report	103	the same
41	Otoukesh [54]	2014	Kidney diseases	Iran	Quantitative, cross-sectional	23,167	the same
42	Otoukesh [55]	2015	Cancer diseases	Iran	Literature review	23,167	the same
43	Etemadifar [56]	2017	MS	Isfahan	Quantitative, cross-sectional	4,536	6
*Iran: mental health*							
44	Sadeghi [57]	2016	Social support	Savojbolagh	Quantitative, cross-sectional	400	the same
45	Dadfar [58]	2015	Mental disorders	Tehran	Quantitative, survey	453	the same
46	Kalantari [59]	2012	Traumatic grief symptoms	Qom	Quantitative, case–control	29 cases 32 controls	the same
47	Mohammadian [60]	2005	Mental disorders	Tehran	Quantitative, survey	453	the same
48	Motamedi [61]	2003	Depression	Kerman	Quantitative, survey	300	the same
49	Kalafi [62]	2002	Mental disorders	Shiraz	Quantitative, survey	81	the same
*Iran: healthcare access & use*							
50	Takbiri [63]	2020	Primary Health Care (PHC)	Tehran	Qualitative, interviews	25	the same
51	Azizi [64]	2019	PHC	Saveh	Qualitative, interviews	18	the same
52	Amiri [65]	2019	Health-seeking behavior	Mashhad	Qualitative, ethnographic	14	the same
53	Mohammmadi [66]	2017	Inequitable care	Tehran	Qualitative, interviews	15	the same
54	Riahi [67]	2016	Health information needs	Iran	Quantitative, survey	483	316
55	Heydari [68]	2016	Health service delivery	Mashhad	Qualitative, interviews	19	the same
56	Roshan-Pajouh [69]	2014	HIV prevention	Mashhad & Varamin	Qualitative, assessment	-	-
57	Dehghan [70]	2013	Immunization coverage	Kerman	Quantitative, cross-sectional	-	-
*Iran: multi aspects of health*							
58	Roozbeh [71]	2018	Health status	Iran	Systematic review	-	-
59	Hosseini Divkolaye [72]	2017	Health challenges	Iran	Systematic review	-	-
60	Shamsi Gooshki [73]	2016	Health & social justice	Iran	Systematic review	-	-
*Germany: physical health*							
61	Begemann [74]	2020	Environmental risk	Various districts	Quantitative, survey	133	27
62	Goodman [75]	2018	Health needs	Saxony	Quantitative, cross-sectional	2,753	402
63	Haesler [76]	2018	Antibiotic resistance	Neumünster	Quantitative, case–control	506 cases 100 controls	95
64	Reinheimer [77]	2017	Multidrug-resistant organisms	Frankfurt	Quantitative, screening	117	30
65	Reinheimer [78]	2016	Multidrug-resistant organisms	Frankfurt	Quantitative, screening	143	29
66	Wollina [79]	2016	Dermatologic challenges	Dresden	Quantitative, cross-sectional	1,100	165
67	Diel [80]	2004	TB	Hamburg	Epidemiological	12,751	6,206
*Germany: mental health*							
68	Mobashery [81]	2020	Depression	Berlin & Hamburg	Qualitative, interviews	100	50
69	Croissant [82]	2020	Endocannabinoid concentrations & mental health	Leipzig	Quantitative, survey	93	53
70	Mueller [83]	2019	Stress	Bavaria	Quantitative, survey	98	53
71	Walther [84]	2019	Psychological distress	Germany	Quantitative, survey	2,639	323
*Germany: healthcare access & use*							
72	Borgschulte [85]	2018	Healthcare provision	Cologne	Mixed-methods (administrative data + interview)	Qualitative: 16	the same
73	Bauhoff [86]	2018	Utilizations & costs of care	SchleswigHolstein & North Rhine-Westphalia	Report	3,639	669
*Germany: multi aspects of health*							
74	Biddle [87]	2019	Health monitoring	Baden-Wuerttemberg	Quantitative, survey	412	80
75	Marquardt [88]	2016	Health status and disease burden	Bielefeld	Quantitative, survey	102	19

### Health condition of Afghan refugees in Iran

Results of the current systematic review revealed that of the 60 health studies on Afghan refugee population in Iran, the majority of studies was in the area of physical health whereas studies of mental health and healthcare access/use were less. According to the results, both genders, all types of ethnicities, adults, and children, were subject to the studies.

#### Physical health

In case of Iran, forty-three eligible physical health studies were reviewed mainly covering the areas of communicable and non-communicable diseases (NCDs), food security, and reproductive health including the period of 2004–2020. Sample sizes of the reviewed studies were ranging from 4 up to 23,167 Afghans.

Communicable diseases: According to our review and the other Iranian systematic reviews and meta-analysis [31, 71–73], the major infectious diseases among Afghan population were Tuberculosis (TB) [44–52], Malaria [42], Cholera [32], Crimean-Congo hemorrhagic fever [34], Leishmaniosis [22, 23], and Hepatitis B [35, 36]. In addition, several HIV- and drug use- related studies had been done [41]. According to a cross-sectional study, among identified HIV-positive Afghans who were mostly male, young, and unemployed, use of Heroin Crack was prevalent and they were likely to be engaged with high-risk behaviors (e.g. shared injection materials or having unprotected sex) compared to the other drug users [38]. Peer education seems to have been an effective and easy-to-apply method to increase knowledge and improve attitudes about HIV at least among street children, while it seemed to be less efficient concerning the long-term reduction in risky sexual behavior [39]. Via a mixed-method exploratory study on substance use, it was observed that Afghan households had at least one adult member using illegal drugs (4.2% out of 2,034 Afghans) and substance use had notable statistical associations with illiteracy, being undocumented, living in slum areas, and poverty [37].

Non-communicable diseases: Kidney diseases [54] and transplantation [53], cancers [55], Multiple Sclerosis (MS) [56], Food insecurity, and nutritional status [25–29] were mostly studied in this area. In general, the referral rate to hospitals for female Afghans and Afghans over 60 years old was higher (compared to male and younger Afghans) and mainly due to ophthalmic diseases, neoplasms, and nephropathies [24]. Two retrospective cross-sectional studies were done on kidney diseases and transplantation. According to one study, both graft and patient survival rates for Afghan patients were good and comparable to Iranian patients [53], another one showed that the most common referral for females and males was end-stage renal disease [54]. According to one study, cancer diagnoses were the cause for 3,083 of 23,152 total referrals and the most common referral for females and males were malignant neoplasms of lymphatic and hematopoietic tissue (34.2%) [55]. MS disease was of lower prevalence among Afghans residents compared to the overall prevalence of the respective province [56].

Five cross-sectional studies on food security and nutritional status were reviewed with samples ranging from 150 to 606 including females and children [25–29]. Three studies showed a high prevalence of food insecurity specifically for females. The income of the families’ heads and the number of male children were associated with food security and diet diversity. Food insecurity was not only significantly more prevalent among female-headed households [26], but also in those families whose head had a lower level of education and socioeconomic status, belonged to the Sunni section, and those without legal residential status [28]. Two studies were done regarding underweight/obesity; one showed that among 606 Afghan children,16% were underweight and 2.8% were overweight [89], the other study that showed that) the prevalence of underweight and wasting was remarkable among children, indicating malnutrition [27] while about 58% of females were overweight/obese (among 414 registered Afghan refugee households). Several mostly quantitative studies had been done assessing reproductive health of Afghan females and infants’ health [14–20]. Some demonstrated a high prevalence of preterm birth and low birth weight of infants. According to one study, race and different factors including lack of proper insurance, availability of health services and a lower socioeconomic status seemed to be connected with preterm birth increase [17]. Suboptimal care (in comparison to Iranian mothers) and Maternal Near Miss (MNM) were reported as well. MNM seemed to be mainly caused by late recognition, misdiagnosis, unfitting care plan, postponements in care-seeking and expensive care services [15]. According to one study, reducing unnecessary cesarean sections and providing insurance coverage for emergency obstetric care might improve maternal and perinatal outcomes [16]. Regarding pregnancy, some Sunni Afghan males seemed to be against using contraception due to religious thoughts [21].

#### Mental health

Of the sixty reviewed health studies, only six merely quantitative studies [57, 62] were done on mental health of Afghan refugees with samples ranging from 29 to 453 Afghans. According to one, the prevalence of mental disorders in Tehran as the capital of Iran was high and mainly females were suffering from mental disorders. Prevalences of social dysfunction, anxiety, and somatic symptoms were higher than depression [60]. Further, severe depression was reported as the most common sign of mental disorders among Afghan refugees in Tehran [58]. A significant correlation was found between mental disorders and characteristics like social support [57] or type of residence [60] but not age [61], marital status [61, 62], or family size [60]. According to an empirical study, a group intervention program was effective for bereaved children and adolescents after experiencing disasters [60].

#### Healthcare access and use

Studies in this area, like the mental health studies, were rare (eight studies) with a sample range of 14 to 316 Afghans [63–70]. According to an ethnographic study, healthcare management for female Afghan refugees carried several obstacles many of which referred the traditions, patriarchy and culture [65]. Two qualitative studies explored the experience of receiving maternal care among Afghan mothers. According to one, the main experiences of Afghan mothers were maltreatment in the form of discrimination and inadequate medical attention. Poor communication with caregivers, financial restrictions, lack of health insurance, and low literacy were other barriers [66]. The second study addressed the importance of health education and its positive changes in raising awareness of refugees on high-risk behaviors reduction as well as increasing HIV testing and identification of patients, and referring them to treatment centers [69].

Afghan refugees had several challenges in every stage of PHC delivery including high service cost and lack of insurance coverage, language barriers, healthcare provider's behavior, delay in getting service in PHC centers [63, 64]. In addition, documented Afghan families who were served by local health clinics usually did not have medical records in these clinics caused by continuing migration behavior due to job-seeking purposes or illegal status. Afghan patients who are illegal may be treated, but no records are kept for them [21]. According to a cross-sectional study on Afghan children, vaccine coverage of BCG, Polio (at birth and round 3), DTP and Hepatitis B (both round 3) and MMR (round 1), was more than 95% which is not different from the coverage of Iranian children but significantly higher than that of Afghan children living in Afghanistan [70].

### Health condition of Afghan refugees in Germany

Results of the current study showed that refugee health studies in Germany were less compared to Iran. Besides, the health status of the Afghan refugee population residing in Germany had mostly been studied including refugees and migrants from a wide range of countries. Hence in the current systematic review, we tried to stay focused on reviewing the related literature with the identifiable share of Afghan refugees in the samples and findings.

#### Physical health

The reviewed physical health studies on Afghan refugees residing in Germany were mostly quantitative and mainly in the areas of TB, antibiotics and drug resistance, dermatologic care, and the assessment of the overall health condition with samples ranging from 16 to 6,206 Afghan cases.

Communicable diseases: According to a study in a primary care refugee clinic (with 402 Afghan patients), the most frequent diagnoses were respiratory, miscellaneous symptoms and otherwise not classified ailments, infection, musculoskeletal or connective tissue disease or gastrointestinal symptoms, and injuries [75]. Another descriptive study in a temporary emergency refugee camp (with 165 Afghans) indicated that the majority of patients attended the clinic with communicable diseases such as bacterial or viral infections. Wounds and chronic inflammatory diseases were rather uncommon [79]. Besides, Gram-negative bacteria colonized 60.8% of 143 refugee patients (including 29 Afghans) admitted to a hospital exceeding that of German resident patients four-fold [78]. In the case of multidrug-resistant and antibiotic resistance, the fecal microbiota of refugees (500 refugees, 23% Afghans and 100 German controls) was substantially different from that of German residents. The majority of refugees carried five or more antibiotic resistance genes whereas the majority of German controls carried three or fewer [76]. Only a minority of TB cases among foreign-born individuals were identified by screening of asylum seekers [80]. Of 412 refugees (including 80 Afghans) long-lasting limitations, bad or very bad overall health, pain, and chronic illness were reported in adults. In 12 months, 52% used primary care and 37% specialist care with 31% unmet needs for primary and 32% for specialist care [87].

Non-communicable diseases: Of 102 unaccompanied asylum-seeking adolescents (including 19 Afghans) a high prevalence of infections, mental illness and iron deficiency anemia and a very low prevalence of NCDs were found [88]. Of 133 young refugees (including 27 Afghans) who were screened in another study, 42.8% had more than three risk factors. According to this study, young refugees arriving in hosting countries with alarming risk burden may be highly vulnerable towards development of neuropsychiatric disorder, behavioral abnormalities, and global functional deficits [74].

#### Mental health

Compared to Iran, almost the same number of studies addressed the mental health status of Afghan refugees in Germany. However, only few studies had a clear and independent sample size of refugees from Afghanistan, and most were emphasizing on depression and anxiety issues. A qualitative study explored the relationship between perceived causes of depression and desire for social distance. According to it, for depressed individuals there were a desire for a social distance. However, a higher number of years spent in Germany was associated with less desire for social distance [81]. According to a large screening (2,639 adult refugees, 323 Afghans), almost half of the population was affected by psychological distress, 10.9% were positive for severe distress and the risk of distress was particularly high for Afghans [84]. Another survey (98 individuals, 53 Afghans) reported a high level of psychological distress and large numbers of potentially traumatic events. The total number of traumatic experiences was identified to be the strongest predictor for depression, anxiety, and Post Traumatic Stress Disorder. Lower individual resources, lower social support in the destination country, and poorer German language skills were associated with a higher level of psychological distress [83].

#### Healthcare access and use

Likewise Iran’s health studies on healthcare access and utilization by Afghan refugees, studies on this category were rare in Germany. Performed studies recommended more health screenings, prevention, and the leverage of barriers to health service access for refugees and unaccompanied asylum-seekers. A dental screening evaluated the oral health of refugees and estimated the costs of oral care among 102 refugees (including 19 Afghans). Around half of the study sample suffered from toothache. The average cost of conservative treatment was calculated to be 205.86 EUR per person, and the average price of prosthetic treatment was calculated to be 588.00 EUR [90]. According to a study on morbidity, utilization, and costs of care of 3,639 asylum-seekers (including 669 Afghans), refugees had more hospital and emergency department admissions including admissions which could have been avoided by prevention or good outpatient care. The average costs were 10% higher than for the normally insured individuals. However, there was considerable dissimilarity in costs by country of origin [86].

## Discussion

Afghans are the main refugee population in Iran who first took asylum in the country over 50 years ago. In Germany, the majority of recent refugees and asylum-seekers come from Syria, Afghanistan, and Iraq. This systematic review focused on those refugee health studies that were mainly done on Afghan refugee populations—the common refugee group between the two countries. Several studies in Iran and Germany had been performed to evaluate the health condition of Afghan refugees. In Germany, since 2014, health studies on refugees and migrants received more attention due to a high wave of asylum-seekers entering mainly from the Middle East, particularly from Syria, to take asylum in the country [91, 92]. Despite several studies on the health of Afghan refugees, the overall health status of this population was not clearly systematically researched neither in Iran nor in Germany. The findings of our systematic review of the past studies revealed health research gaps for this population and provides an overview of the current health condition of Afghan refugees as well as some evidence-based recommendations for improving this population’s health. Concerning both countries, gaps in the so far performed studies of mental health, healthcare access/use were observed with such insufficiencies particularly for Germany. A systematic review of migrants’ health from the perspective of social justice in Iran confirmed these shortages as well [73]. In addition, among German refugee health studies, qualitative studies in the mentioned areas were rare. However, based on the chosen search strategy, some relevant studies might have been missed. For instance, there might be a range of studies without mentioning “Afghan” refugees but reporting health data for this population especially in case of Germany.

German health studies principally explored the health condition of all groups of refugees and asylum-seekers to find certain physical or mental diseases rather than using a stratification of results by country of origin to capture the within-group diversity of refugees. This issue was also criticized by a study mentioned before [93]. According to this study, distinction within the immigrant population (e.g., social and legal status, country of origin, and duration of stay) is essential. To stay focused on the frame of the purpose of the current study, we examined those German health studies merely with the samples of Afghans and at least with independent outcomes for Afghans. However, results were limited and rare. Contrary to the Iranian health studies on Afghan refugees’ physical health, German health studies in this area were fewer and less comprehensive. Our findings showed that several reproductive health studies of female Afghan refugees existed in Iran, but no specific study on the reproductive health of female Afghan refugees in Germany. In Iranian studies in the area of communicable diseases, TB, malaria, cholera, Crimean-Congo hemorrhagic fever, leishmaniosis, and hepatitis B seemed to be the major infectious diseases for the Afghan population. Substance use and related infectious diseases are other issues. In Germany, the prevalence of TB did not seem to be high among all refugees including Afghans. However, the prevalence of iron deficiency anemia, some other infectious diseases (e.g., dermatologic infections), and general antibiotic multidrug resistance were found to be high. In Iran, food insecurity and specific types of cancers (e.g., malignant neoplasms of lymphatic and hematopoietic tissue and the digestive system) were prevalent while in Germany no studies on such diseases were found, neither for other communicable diseases such as HBV and HIV/AIDS.

In the area of mental health, the prevalence of mental disorders varied for Afghan refugees living in different cities of Iran, but in general was found to be high. German studies confirmed both high psychological distress and depression among Afghan refugees. The Iranian studies on the area of healthcare access and use by Afghan refugees demonstrated poor communication with healthcare providers, difficulties with recording refugees’ health data, tradition and culturally related aspects of healthcare-seeking behavior, and some language barriers. German studies primarily were focused on the costs of healthcare and expenditure. In general, the language similarities and cultural closeness to the Iranian population seemed to be an important factor for employment, access, and use of healthcare and the integration of Afghan refugees into the Iranian society.

### Recommendations to improve the health of the Afghan refugee population

Based on the reviewed studies, in the case of TB, early case detection efforts focusing on migrant populations are recommended [51]. Regarding HIV/AIDS among Afghan refugees, studies suggest drug treatment, therapeutic community services, programs such as condom education and distribution, HIV/AIDS education, voluntary counseling and testing, and needle and syringe programs [33, 38, 39, 41]. Another approach would be screening strategies against diseases like HBV [35]. Furthermore, due to the high prevalence of certain cancer diagnoses among refugees, implementing effective screenings and prevention as well as improved health record-keeping of refugees and provision of sustainable funding sources in collaboration with global humanitarian agencies are essential measures [55].

To promote Afghan refugees’ nutritional health and food security, interventional and educational programs, facilitating Afghan refugees’ employment, and improving access to welfare facilities especially for females and children, are recommended [25, 29]. In the case of reproductive health of Afghan females, improving obstetric practice and targeting exact needs during pregnancy may prevent MNM consequences [15]. Another apparent measure would be providing the refugees with health insurance coverage which facilitates financial access to healthcare [54, 93–96]. Further strategies are to improve the level of literacy and awareness in the refugee population and to empower them, with special attention towards females [33].

In general, recommendations for Afghan refugees in both studied countries were comparatively similar. However, due to the different discussed settings (Table 1) and slightly different health issues of Afghans in Iran compared to Germany, some recommendations may quite vary for each of the countries. For instance, culture and language-related barriers are important aspects in Germany whereas in Iran they are not a challenge as both are relatively similar among Iranians and Afghans.

## Conclusions

Finally, on the basis of the current systematic review, future refugee empirical health studies are recommended using a wider lens to comprehensively assess the overall condition of refugees considering associations between health and socio-economic and cultural determinants instead of a one-dimensional approach. This comprehensive approach can be a fundament for future health policies and related measures to improve health care services, quality of living and thus a better integration of refugees in hosting countries. Assisting refugees with language learning, informing and educating them (including health education), facilitating their employment process, and supporting them with psychological services, are recommended.

Further, the authors recommend to appropriately consider the diversity between groups of refugees like country of origin and so on. For instance, Afghans and Syrian refugees both come from a similar geographical location – the Middle East. However, Afghan populations experience living conditions dissimilar and often worse compared to other Middle Eastern countries adversely affecting their health. In refugees’ countries of origin, patterns of diseases, health behaviors and habits (e.g., smoking, nutrition, etc.), cultural habits, and in general living conditions are different exposing people to different and different degrees of health threats. This should be a challenge for the host countries to respond to the diverse health needs of various groups of refugees and thus facilitate their integration to a new living condition.

## Data Availability

Data sharing is applicable upon request.
